# The biotechnological potential of marine bacteria in the novel lineage of *Pseudomonas pertucinogena*

**DOI:** 10.1111/1751-7915.13288

**Published:** 2018-06-25

**Authors:** Alexander Bollinger, Stephan Thies, Nadine Katzke, Karl‐Erich Jaeger

**Affiliations:** ^1^ Institute of Molecular Enzyme Technology Heinrich‐Heine‐University Düsseldorf Forschungszentrum Jülich D‐52425 Jülich Germany; ^2^ Institute of Bio‐ and Geosciences IBG‐1: Biotechnology Forschungszentrum Jülich GmbH D‐52425 Jülich Germany

## Abstract

Marine habitats represent a prolific source for molecules of biotechnological interest. In particular, marine bacteria have attracted attention and were successfully exploited for industrial applications. Recently, a group of *Pseudomonas* species isolated from extreme habitats or living in association with algae or sponges were clustered in the newly established *Pseudomonas pertucinogena* lineage. Remarkably for the predominantly terrestrial genus *Pseudomonas*, more than half (9) of currently 16 species within this lineage were isolated from marine or saline habitats. Unlike other *Pseudomonas* species, they seem to have in common a highly specialized metabolism. Furthermore, the marine members apparently possess the capacity to produce biomolecules of biotechnological interest (e.g. dehalogenases, polyester hydrolases, transaminases). Here, we summarize the knowledge regarding the enzymatic endowment of the marine *Pseudomonas pertucinogena* bacteria and report on a genomic analysis focusing on the presence of genes encoding esterases, dehalogenases, transaminases and secondary metabolites including carbon storage compounds.

## Graphical abstract

The newly established *Pseudomonas pertucinogena* lineage consists of several marine bacterial species which possess the capacity to produce biomolecules of biotechnological interest. Beside biocatalysts including dehalogenases, polyester hydrolases, and transaminases they produce several secondary metabolites and carbon storage compounds.

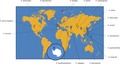

## Introduction

The oceans cover the largest part of the earth's surface and represent one of the most diverse environments on our planet (Venter *et al*., 2004; Armbrust and Palumbi, 2015; Tully *et al*., 2018). Researchers have started to explore this diversity by identification and isolation of biocatalysts and secondary metabolites produced by marine organisms, opening up a novel branch of research and application termed blue biotechnology. Examples comprise not only the famous green fluorescent protein produced by the jellyfish *Aequorea victoria* and its use in innumerable applications including clinical diagnostics and therapeutics (Ohba *et al*., 2013; Enterina *et al*., 2015; Hoffman, 2015), but also antiviral and anticancer compounds isolated from marine sponges (Calcabrini *et al*., 2017), biocatalysts with potential application for the production of pharmaceutical building blocks like solketal (Ferrer *et al*., 2005), and trabectedin (supplied as Yondelis by PharmaMar S.A.) as an example for a chemotherapeutic compound produced by putative endosymbiotic bacteria of the sea squirt *Ecteinascidia turbinate* (Schofield *et al*., 2015). Marine metagenomics and the identification and characterization of isolated marine organisms have led to a huge set of gene and genome sequences, as recently shown by the assembly of more than 2600 draft genomes from data collected during the Tara Oceans circumnavigation expedition (Tully *et al*., 2018), as well as experimental data enabling insights into the biochemical potential of marine habitats and the respective microorganisms (Li and Qin, 2005; Kennedy *et al*., 2008; Popovic *et al*., 2015).

A number of marine bacteria hold great potential for biotechnological applications, for example the marine bacterium *Alcanivorax borkumensis,* which is known to play a key role in bioremediation of oil spills (Schneiker *et al*., 2006). The strain produces a biosurfactant (Yakimov *et al*., 1998) and possesses several genes encoding esterases and monooxygenases of high biotechnological interest (Tchigvintsev *et al*., 2015). Several marine *Acinetobacter sp*. were also shown to produce biosurfactants (Mnif and Ghribi, 2015), for example glycolipoproteins with useful surface‐active properties (Peele *et al*., 2016). An *Enterobacter* species isolated from a shark jaw produces rather uncommon medium‐chain‐length polyhydroxyalkanoates which may have biomedical applications (Wecker *et al*., 2015). Furthermore, a range of novel antibiotics was identified from marine bacteria of diverse sources (Eom *et al*., 2013).

Members of the genus *Pseudomonas* which belongs to the γ‐proteobacteria can colonize diverse habitats and produce useful biomolecules including lipases (Jaeger *et al*., 1996; Liu *et al*., 2017), fluorescent proteins (Torra *et al*., 2015), degradation pathway enzymes (Poblete‐Castro *et al*., 2012), rhamnolipids (Chong and Li, 2017), phenazines (Bilal *et al*., 2017) and a number of heterologous secondary metabolites (Loeschcke and Thies, 2015). The main fraction of *Pseudomonas* species described so far is assigned to terrestrial habitats (Romanenko *et al*., 2005); however, there are also reports of species isolated from marine environments (Baumann *et al*., 1983), e.g. *P. marincola* (Romanenko *et al*., 2008), *P. aeruginosa* (Manwar *et al*., 2004) or *P. glareae* (Romanenko *et al*., 2015).

The huge number of *Pseudomonas* species was phylogenetically distributed into three lineages comprising 13 groups. One and by far the smallest of these lineages, which was only recently established, consists of a single so‐called *Pseudomonas pertucinogena* group. A small number of newly described *Pseudomonas* species with remarkable properties cluster within this lineage (García‐Valdés and Lalucat, 2016; Peix *et al*., 2018). Originally, it consisted of only two species, namely *P. pertucinogena* and *P. denitrificans* (Anzai *et al*., 2000). As the classification of *P*. *denitrificans* is known to be ambiguous (Doudoroff *et al*., 1974), it is difficult to assign respective studies to the correct genus and species; these studies are therefore not considered within this review. The original *P. pertucinogena* group was recently classified as a separate lineage within the genus *Pseudomonas* (Peix *et al*., 2018) comprising nine marine (including the salt lake isolate *P. salina*) and seven non‐marine members (Table 1).

**Table 1:** Bacteria belonging to the *P. pertucinogena* lineage

Species		Habitat^a^	Origin^a^	Temperature range^b^	Salinity range^b^	Accession No^c^	Reference^d^
*P. pertucinogena*		Not recorded, deposit of the ATCC		n.d	n.d		Kawai and Yabuichi (1975)
*P. bauzanensis*		Soil from an industrial site	Bozen, South Tyrol, Italy	5–30°C	0–10%	NZ_FOGN00000000.1 NZ_FOUA00000000.1	Zhang *et al*. (2011)
*P. formosensis*		Food‐waste compost	Taiwan	20–50°C	0–5.0%	NZ_FOYD00000000.1	Lin *et al*. (2013)
*P. populi*		Stems of *Populus euphratica* tree	Khiyik River, China 40°43′22″ N 85°19′18″ E	4–45°C	1–3%		Anwar *et al*. (2016)
*P. saudimassiliensis*		Air samples in an urban environment	Makkah, Saudi Arabia	37°C	n.d.	LM997413.1	Azhar *et al*. (2017)
*P. xiamenensis*		Activated sludge in sewage treatment	Xiamen, China	10–45°C	0–8%		Lai and Shao, 2008;
*P. xinjiangensis*		Desert sand	Xinjiang, China	4–42°C	0–6%	NZ_LT629736.1	Liu *et al*. (2009)
*P. salina* e		Salt lake	Xiaochaidan, China 37°28′53″N 95°30′19″E	4–35°C	0–12.0%		Zhong *et al*. (2015)
*P. aestusnigri* e		Crude oil‐contaminated intertidal sand samples	Spain 42°46′ 29.27″ N 9°7′27.08″ W	18–37°C	2–12.5%	NZ_NBYK00000000.1	Sánchez *et al*. (2014), Gomila *et al*. (2017b)
*P. litoralis* e		Mediterranean seawater	Spain 40°27′24″N 0°31′36″E	15–37°C	0–15%	NZ_LT629748.1	Pascual *et al*. (2012)
*P. oceani* e		Deep‐sea (1350 m)	Okinawa Trough, Pacific Ocean	4–41°C	0–10%	NZ_PPSK00000000.1	Wang and Sun (2016), García‐Valdés *et al*. (2018)
*P. pachastrellae* e		Sponge *Pachastrella*	Philippine Sea	7–41°C	0–10%	NZ_MUBC00000000.1	Romanenko *et al*. (2005), Gomila *et al*. (2017a)
*P. pelagia* e	CL‐AP6 (type strain)	Antarctic green algae *Pyramimonas gelidicola* co‐culture	Antarctic Ocean	4–33°C	0.5–8%	NZ_AROI00000000.1	Hwang *et al*. (2009), Koh *et al*. (2013)
	58	Artic fjord	Norway, Ny Alesund			NZ_NWMT00000000.1	
*P. sabulinigri* e		Black beach sand	Soesoggak, Jeju Island, Korea	4–37°C	0–10%	NZ_LT629763.1	Kim *et al*. (2009)
*P. salegens* e		Aquatic plants of saline wetland	Gomishan saline wetland, Iran 37°03′N 54°01′E	4–35°C	1–10%	NZ_LT629787.1	Amoozegar *et al*. (2014)
*P. profundi* e		Deep‐sea (1000 m)	Pacific Ocean, Mariana Trench 11°23.152′N 142° 29.062′E	4–40°C	0–10%		Sun *et al*. (2018)

a Environment from which the species was isolated (Habitat) and geographical origin of the sample (Origin) as stated in the type strain description.

b As stated in the respective type strain description.

c Accession numbers of GenBank/RefSeq entries for the genomes or, in cases of draft genome sequences, the accession number of the respective master entry.

d References for original descriptions and, if applicable, genome announcements.

S. Thies and A. Bollinger, unpublished data.

e Marine isolates.

Remarkably, enzymes from these bacteria were mentioned in different studies focusing on the bioinformatics identification of novel biocatalysts relevant for biotechnology applications, although most of the relevant species or genome sequences have been described only recently. We summarize here reports on biotechnologically relevant biomolecules produced by marine specimens of the *P. pertucinogena* lineage and describe their respective habitats with prevailing harsh environmental conditions. Based on the findings reported, we have examined the genetic capabilities of this group of bacteria to potentially produce biotechnologically relevant enzymes including polyester hydrolases, rare dehalogenases, ω‐transaminases as well as secondary metabolites and carbon storage compounds. Apparently, bacteria of the *P. pertucinogena* lineage have the potential to produce such biotechnologically relevant biomolecules; however, this has for each case to be experimentally validated.

## The *Pseudomonas pertucinogena* lineage

**Figure 1:**
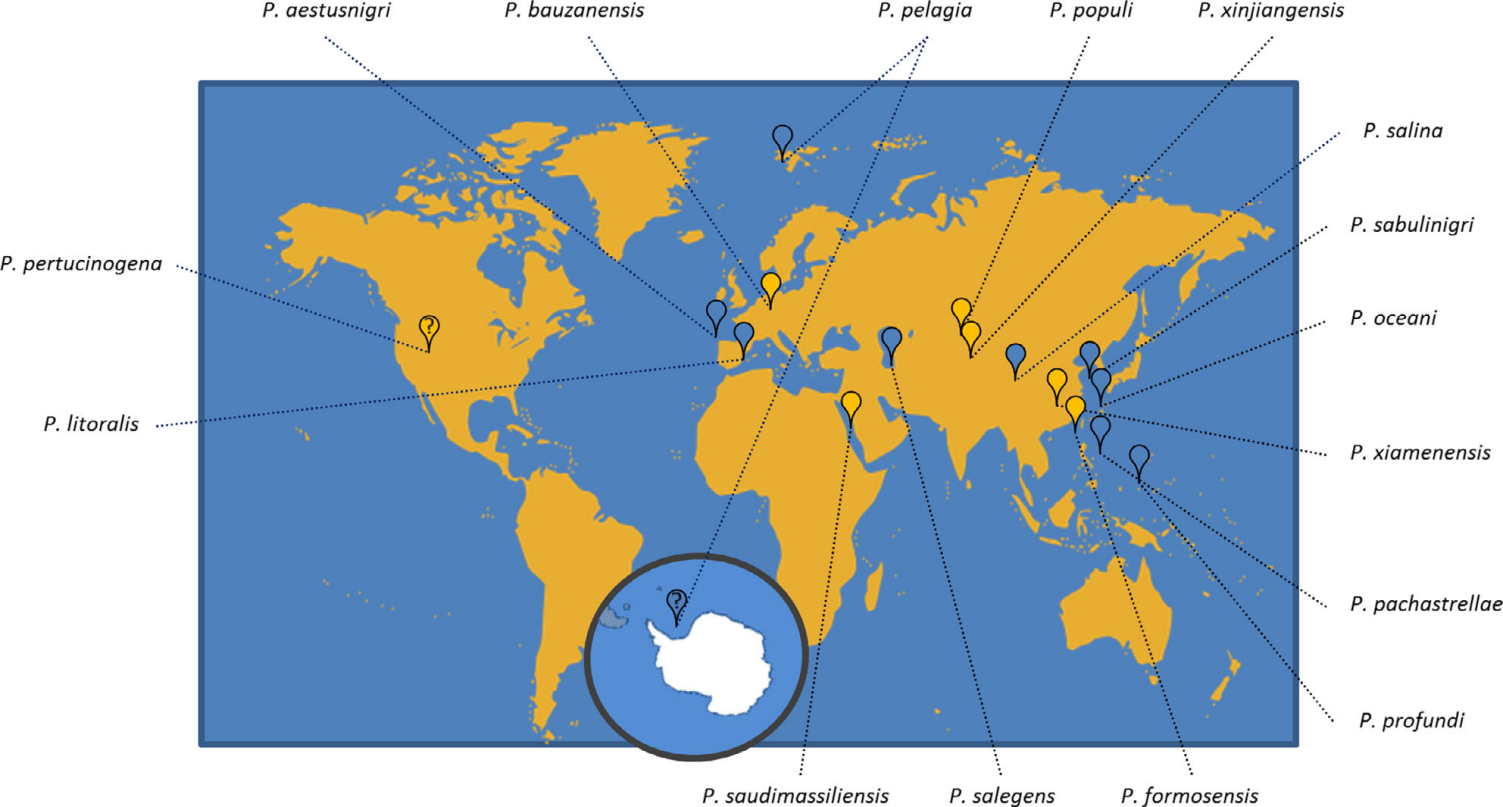
Geographical distribution of 16 known bacterial species clustering in the *P. pertucinogena* lineage. Marine habitats are indicated by blue symbols, ambiguous description of the sampling site is indicated by a question mark. The Antarctic continent is pictured in a circle.

Representatives of the genus *Pseudomonas* are generally equipped with a versatile metabolism that is reflected by a rather large genome with sizes ranging from 4.1 (for some *P. stutzeri* strains) to more than 6 Mbp (e.g. for *P. syringae*,* P. aeruginosa*,* P. putida*,* P. protegens* and *P. fluorescens*). Hilker *et al*. reported a core genome consisting of more than 4000 open reading frames after analysing the genome sequences of 20 different *P. aeruginosa* strains (Hilker *et al*., 2015). Similar dimensions were shown for *P. putida*; the core genome consists of at least 3386 genes and the average genome size is reported with about 6 Mbp (Udaondo *et al*., 2016; Lopes *et al*., 2018). An analysis of 76 newly isolated fluorescent *Pseudomonas* strains from tropical soil showed more than 5500 coding sequences per genome and an average genome size of more than 6 Mbp for the newly assembled genomes (Lopes *et al*., 2018). In contrast, all members of the *P. pertucinogena* lineage possess, as far as known, a comparably small genome of less than 4 Mbp (Table 1, column 6), coding for about 3500 genes. These bacteria were isolated from diverse habitats, for example in association with marine sponges (Romanenko *et al*., 2005), from the air (Azhar *et al*., 2017), deep‐sea sediments (Wang and Sun, 2016) or heavy metal contaminated soil (Zhang *et al*., 2011) and they are distributed over a large geographical area (Fig. 1). Currently, only 16 species are assigned to this lineage, with about half of them attributed to marine environments (Table 1, marked with superscript f). Most of these species were first described during the last 10 years, but the lineage is likely to be further extended in the near future, e.g. by 16 metal resistant endophytic bacteria which appear to be near relatives of *P. sabulinigri* isolated from marshlands (Rocha *et al*., 2016).

Many of the marine species of the *P. pertucinogena* lineage are adapted to cold environments, with reported growth at temperatures below 15°C and tolerance for moderate salt concentrations, surely related to their marine living conditions. Some species were described to live in association with aquatic plants, algae or sponges. As an example for such a symbiotic relationship, *P. pelagia* is discussed to depend on its host's protection against freezing to survive under the harsh conditions in the Antarctic Ocean, as antifreeze activity of the bacterium itself was not observed (Koh *et al*., 2013). Aside from the colonization of algae, plants and sponge surfaces, contaminated environments seem to be a preferred habitat for these marine bacteria, including endophytes isolated from a heavy metal accumulating plant at a contaminated salt marshland (Rocha *et al*., 2016) and crude oil‐contamination sites (Lamendella *et al*., 2014 and Sánchez *et al*., 2014). The ability to degrade different hydrocarbons, the major constituents of crude oil, was proposed for *P. aestusnigri*, which possesses genes encoding phenol hydroxylase for degradation of aromatic hydrocarbons as well as an alkane‐1‐monooxygenase for aliphatic alkane degradation (Gomila *et al*., 2017b).

Bacteria belonging to the genus *Pseudomonas* are generally well known for their versatile metabolism. The metabolic flexibility of Pseudomonads is reflected by their ability to use for growth a range of different carbon sources including carbohydrates, organic acids, alcohols, alkanes and most amino acids (Palleroni, 1984; Daniels *et al*., 2010). A comprehensive phenomics analysis of *P. putida* strain DOT‐T1E revealed a complex hierarchical network regulating the utilization of different carbon, nitrogen or sulfur sources (Daniels *et al*., 2010). In contrast, members of the novel *P. pertucinogena* lineage seem to be rather limited with respect to the spectrum of utilizable carbon sources. Sánchez *et al*. described eight species of the *P. pertucinogena* group which utilize short chain carbonic acids, e.g. lactate or propionate and few amino acids, whereas they fail to utilize glucose as well as 75–80% of all tested carbon sources provided by standard phenotyping assays (Sánchez *et al*., 2014). Undoubtedly, additional studies are needed to explore the metabolism of these bacteria in more detail. However, the currently available data suggest that strains of the *P. pertucinogena* group may constitute an exception of niche‐adapted specialists within the genus *Pseudomonas*, as already suggested by the specific sites of isolation and further corroborated by their small genomes and their limited metabolic flexibility. The psychrophilic and moderate halophilic habitats indeed suggest potential for a variety of biotechnological applications of the bacteria themselves, but also their enzymes (Cavicchioli *et al*., 2011; Cafaro *et al*., 2013; Yin *et al*., 2015; Danso *et al*., 2018).

Enzymes produced by bacteria of the *P. pertucinogena* lineage have been mentioned by several studies, in particular those useful for polymer degradation and synthesis of chiral molecules (Schallmey *et al*., 2014; Haernvall *et al*., 2017, 2018). Enzyme coding genes were identified by mining of sequence data and subsequently expressed in heterologous hosts to prove their functionality. The results indicate that *in silico* mining of *P. pertucinogenae* genomes indeed constitutes a suitable strategy to assess the biotechnological potential of this currently still small group of *Pseudomonas* species. Hence, we have analysed the genomes of seven species of the marine *P. pertucinogena* lineage with respect to their capacity to produce biotechnologically relevant enzymes and compounds. In general, BLASTP (Altschul *et al*., 1997) was applied to identify homologues of proteins reported in literature taking at least 40% identity to the query sequence and query coverage of at least 85 % as lower borders for selection. However, as a number of closely related species were analysed, the sequence homology of homologous proteins was significantly higher, as stated in the respective paragraphs. These analyses revealed a considerable number of genes encoding useful enzymes as well as the potential to synthesize secondary metabolites and storage compounds (Table 2).

**Table 2:** The catalytic and biosynthetic potential of marine *P. pertucinogena* bacteria. Genome sequences were analysed with different bioinformatics tools for the presence of polyester hydrolases (PE hydrolase), halohydrin dehalogenases (HHDH and HheD12), ω‐transaminases (ω‐TA), flavin‐binding fluorescent proteins (FbFPp), polyhydroxyalkanoates (PHA) and ectoin synthesis clusters (Ectoin)

Species	Strain	PE hydrolase^a^	HHDH^b^	ω‐TA^c^	FbFP^d^	PHA^e^	Ectoin^e^
*P. aestusnigri* f	VGXO14	WP_088276085.1	WP_088273591.1	WP_088276225.1 WP_088273722.1 WP_088276261.1	WP_088273209.1	Yes	Yes
*P. litoralis* f	2SM5	WP_090272969.1	–	WP_090274676.1 WP_090275926.1	–	No	Yes
*P. pachastrellae* f	CCUG 46540	WP_083724990.1	WP_083723433.1	WP_083728130.1	WP_083728464.1	Yes	Yes
*P. pelagia* f	58	WP_096345769.1	WP_096348266.1	WP_096346315.1 WP_096346382.1	WP_096345677.1	Yes	Yes
	CL‐AP6	WP_022964382.1	WP_022962804.1 (HheD12)	WP_022961575.1 WP_022964449.1 WP_022963892.1	WP_022961159.1	Yes	Yes
*P. sabulinigri* f	JCM 14963	WP_092287377.1	WP_092284942.1	WP_092286338.1 WP_092286396.1	WP_092288528.1	Yes	Yes
*P. salegens* f	CECT 8338	WP_092388080.1	WP_092387787.1	WP_092388656.1 WP_092389204.1	WP_092383819.1	Yes	Yes
*P. oceani* f	DSM 100277	WP_104736494.1	WP_104737909.1	WP_104738025.1 WP_104739904.1 WP_104737746.1	WP_104739045.1	Yes	Yes

a Proteins with at least 70% identity to the polyester hydrolase PpelaLip from *P. pelagia* (Haernvall *et al*., 2017).

b Proteins with high similarity to *P. pelagia* HheD12 (Schallmey *et al*., 2014).

c Proteins with at least 40% identity to selected known ω‐TAs as query sequence and a query coverage of at least 90%.

d Proteins with identities >60% to PpSB1‐LOV (NP_746738.1), identified by BLASTP (Altschul *et al*., 1997).

e Presence of a complete metabolite synthesis cluster predicted by the antiSMASH pipeline (Weber *et al*., 2015; Blin *et al*., 2017).

f Marine isolates.

## Biocatalysts

Enzymes are applied for a large number of different biotechnological applications, but increasingly also as (often enantioselective) biocatalysts in synthetic organic chemistry driving the development of green and sustainable processes (Sheldon and Woodley, 2018). Here, enzymes to be obtained from bacteria of the *P. pertucinogena* lineage can significantly contribute, in particular polyester hydrolases, dehalogenases and transaminases.

### Polyester hydrolases

Carboxylic ester hydrolases (EC 3.1.1.) represent an important group of biocatalysts for industrial applications in a wide range of different sectors, like the pulp and paper, the pharmaceutical and the food industry (Singh *et al*., 2016). They catalyse both the hydrolysis and the synthesis of esters, often with high enantioselectivity (Casas‐Godoy *et al*., 2012). During the last decades, the degradation of polyester compounds became more and more important due to the increasing environmental pollution with non‐biodegradable polyesters such as polyethyleneterephthalate (PET) (Narancic and O'Connor, 2017). As this synthetic polyester cannot be properly recycled, more than 70% of the total plastic packaging waste may ultimately enter the food chain through inadequately treated waste water, the oceans and subsequently marine micro‐ and macroorganisms (Wei and Zimmermann, 2017).

The potential of *P. pelagia*, a member of the *P. pertucinogena* lineage, to effectively degrade ionic phthalic acid‐based polyesters was shown recently (Haernvall *et al*., 2017). The respective biocatalyst is a putatively secreted lipase designated as PpelaLip, which was identified by a homology guided sequence search of different extracellular hydrolases from *Pseudomonas sp*. using as a template the amino acid sequence of the *Thermobifida cellulosilytica* cutinase (Thc_Cut1), an enzyme known to efficiently hydrolyse different polyesters. After successful recombinant production and purification, the activity of the enzyme was experimentally demonstrated with different polyester substrates (Haernvall *et al*., 2017). In a successive study, the applicability of this biocatalyst for wastewater treatment was shown (Haernvall *et al*., 2018). We also performed a homology search with BLASTP (Altschul *et al*., 1997) using *P. pelagia* lipase PpelaLip as a query against all published genome sequences from the *P. pertucinogena* lineage (Table 1, column 6) and identified putative proteins with 70–80% sequence identity. Interestingly, these PpelaLip homologous enzymes are not unique within the marine bacteria of the *P. pertucinogena* lineage, as we have identified homologs in all *P. pertucinogena* species (Tables 2 and 3). Apparently, representative species of the *P. aeruginosa* and *P. fluorescens* lineages also encode such enzymes; however, the overall similarity is low indicating that this type of putative polymer‐degrading enzyme represents a distinct characteristic of the *P. pertucinogena* bacteria (Table 3). In a recent study of a PETase from *Ideonella sakaiensis* (Yoshida *et al*., 2016), the classification of PETases in three groups was suggested based on amino acid sequence alignments and putative enzymes from *P. sabulinigri*,* P. pachastrellae* and *P. litoralis* were grouped into type IIa of PET‐degrading enzymes (Joo *et al*., 2018).

**Table 3:** Amino acid sequence homology expressed as identity in percentage to the known polyester hydrolase PpelaLip (Haernvall *et al*., 2017) from *P. pelagia* strain CLAP6 as identified using BLASTP (Altschul *et al*., 1997)

	No.	Organism	Strain	Identity in %	Protein ID
Marine	1	*P. pelagia*	CL‐AP6	100	WP_022964382
	2	*P. pelagia*	58	80	WP_096345769
	3	*P. aestusnigri*	VGXO14	74	WP_088276085
	4	*P. litoralis*	2SM5	73	WP_090272969
	5	*P. pachastrellae*	JCM 12285	74	WP_083724990
	6	*P. sabulinigri*	JCM 14963	72	WP_092287377
	7	*P. sabulinigri*	JCM 14963	71	WP_092287378
	8	*P. salegens*	CECT 8338	72	WP_092388080
	9	*P. salegens*	CECT 8338	70	WP_092388077
	10	*P. oceani*	DSM 100277	74	WP_104736494
Not marine	11	*P. formosensis*	JCM 18415	73	WP_090538641
	12	*P. saudiamassiliensis*	12M76	73	WP_044499735
	13	*P. xinjiangensis*	NRRL B‐51270	72	SDS09569
	14	*P. xinjiangensis*	NRRL B‐51270	76	WP_093397383
Other *Pseudomonas* species	15	*P. syringae*	ICMP13650	31	KPW53696
	16	*P. aeruginosa*	PA01	28	WP_003143191
	17	*P. putida*	ATH‐43	27	WP_046786320
	18	*P. protegens*	4	24	WP_102863500
	19	*P. stutzeri*	28a39	52	WP_102852227
	20	*P. fluorescens*	C3	29	WP_046049461

### Dehalogenases

Dehalogenases catalyse the cleavage of carbon–halogen bonds and have potential applications in the chemical industry and for detoxification (Kurihara and Esaki, 2008). They are used for organic synthesis of optically pure building blocks, recycling of by‐products from chemical processes, bioremediation and biosensing of toxic pollutants (Koudelakova *et al*., 2013). Halohydrin dehalogenases (HHDH) represent a particularly interesting type of dehalogenases which naturally catalyse the dehalogenation of haloalcohols with the formation of the corresponding epoxides. In the reverse reaction, i.e. opening of the epoxide ring, they behave promiscuous accepting a wide range of nucleophiles such as azide, cyanide or nitrite enabling the synthesis of a wide range of chiral molecules (Schallmey *et al*., 2014; Koopmeiners *et al*., 2016). Schallmey *et al*. recently developed a bioinformatics pipeline to uncover these rare enzymes within sequence data sets and successfully accessed selected hits by heterologous expression and subsequent demonstration of HHDH activity. During this study, an HHDH was identified in the *P. pelagia* CL‐AP6 genome and assigned to subgroup D, designated as HheD12 (Schallmey *et al*., 2014). This same study unveiled that other *Pseudomonas* species did not encode HHDHs; and only five of 43 reported enzymes originate from γ‐proteobacteria. A BLASTP analysis of the available genome data within the *P. pertucinogena* lineage with the *P. pelagia* CL‐AP6 enzyme (WP_022962804.1) as a query revealed hits with identities between 91% (*P. pelagia* strain 58) and 68% (*P. salegens*) in all marine species with the exception of *P. litoralis* (Table 2). Within the terrestrial isolates, only the *P. xinjiangensis* genome encoded such an enzyme (identity 77%). This suggests an important role for these enzymes especially in marine environments.

### ω‐Transaminases

Chiral amines are valuable building blocks for a variety of compounds produced by the chemical and pharmaceutical industries. For pharmaceuticals, an estimated share of 40% contains at least one amine functionality (Kelly *et al*., 2018). While several options for enzymatic chiral amine production exist, asymmetric synthesis by ω‐transaminases (ω‐TA) is greatly preferred as the theoretical yield is 100% (Cassimjee *et al*., 2010; Koszelewski *et al*., 2010). Most TAs need pyridoxal‐5′‐phosphate as a cofactor and catalyse the asymmetric synthesis of chiral amines by transferring an amino group from an aminated donor to various carbonyl compounds (Savile *et al*., 2010; Börner *et al*., 2017; Guo and Berglund, 2017). A diverse array of reactions is reported towards the synthesis of pharmaceuticals or pharmaceutical intermediates involving ω‐TAs by both asymmetric synthesis and kinetic resolution (Kelly *et al*., 2018). Thus, the identification of novel enzymes with ω‐transaminase activity is of high importance for both science and industry.

*Pseudomonas* sp. appear to be a promising source for ω‐transaminases (Wilding *et al*., 2015; Poehlein *et al*., 2017; Wu *et al*., 2017). Recently, an ω‐transaminase which most probably originates from *P. sabulinigri* was discovered during an activity‐based screening of a metagenomic library which originated from a polluted harbour site in Ancona, Italy (M. Ferrer, personal communication). While *P. sabulinigri* was originally identified in samples from Korean beach (Table 1), another report on closely related specimen discovered in a metalloid polluted salt marsh in the northwest coast of Portugal (Rocha *et al*., 2016) hints at the potential of this *Pseudomonas* strain to thrive in contaminated environments.

Based on this finding, a homology search with the BLASTP tool was performed to gain insights into the potential of marine representatives of the *P. pertucinogena* lineage for the production of ω‐transaminases following a strategy reported earlier (Mathew and Yun, 2012). After searching the genome sequences of *P. aestusnigri*,* P. litoralis*,* P. sabulinigri*,* P. salegens*,* P. pelagia* strain 58, *P. pelagia* strain CL‐AP6, *P. pachastrellae* and *P. oceani*, the results were filtered for hits with at least 40% identity to the query sequence and query coverage of at least 90%.

The amino acid sequences of the four recently characterized (*S*)‐selective ω‐transaminases from *P. putida* (Wu *et al*., 2017) were used as the main set of queries. The respective enzymes belong to differing families of transaminases, namely the 4‐aminobutyrate pyruvate aminotransferase family (EC 2.6.1.96, BAN53958.1), beta‐alanine pyruvate transaminase family (EC 2.6.1.18, BAN52522.1), aspartate aminotransferase family (EC 2.6.1.1, BAN55495.1) and putrescine‐pyruvate aminotransferase (EC 2.6.1.113, BAN57107.1). BAN52522.1, which is identical to the enzyme used for resolving a crystal structure (PDB 3A8U), delivered one hit with *P. litoralis*,* P. sabulinigri*,* P. salegens*,* P. pelagia* (both strains) and *P. pachastrellae*, and two hits with *P. aestusnigri* and *P. oceani*. Additional searches with sequences of BAN53958.1, BAN55495.1 and BAN57107.1 each returned the same enzyme sequence in every examined genome, as well as a second hit in the genome of *P. pelagia* CL‐AP6. All hits have been annotated as aspartate aminotransferase family proteins according to the NCBI database.

Additional homology searches with sequences of (*S*)‐selective ω‐transaminases from *Ruegeria pomeroyi*,* Vibrio fluvialis* and *Chromobacterium violaceum,* all attributed to the aspartate aminotransferase family, returned hits with the same amino acid sequences as the aforementioned (*S*)‐selective transaminases from *P. putida*, with identities up to 60%. Homology searches with (*R*)‐selective transaminases from *Aspergillus fumigatus*,* Aspergillus fisheri*,* Fusarium graminearum* and *Arthrobacter* sp. according to Pavlidis *et al*. (2016) delivered no results within the search parameters. Conclusively, every searched genome contains at least two structurally different putative ω‐transaminases, *P. aestusnigri*,* P. oceani* and *P. pelagia* CL‐AP6 contain three such candidate enzymes (Table 2).

### Flavin‐binding fluorescent proteins

Flavin‐binding fluorescent proteins (FbFPs) were developed as reporter proteins which constitute an oxygen‐independent alternative to the family of green fluorescent proteins. They are derived from blue light photoreceptors of the L(ight)‐O(xygen)‐V(oltage) domain family (Drepper *et al*., 2013; Buckley *et al*., 2015). Besides their O_2_ independence, FbFPs are small proteins (M_r_: 12–16 kDa) and exhibit fast folding kinetics; thus, they are valuable reporter proteins for quantitative real‐time analysis of different bio(techno)logic processes (Potzkei *et al*., 2012; Rupprecht *et al*., 2017). Commonly, LOV photoreceptors consist of LOV domains fused to various effector domains which are activated by conformational changes of the LOV domain in response to a light stimulus. However, predominantly among bacteria, the so‐called short LOV proteins only consisting of the light‐perceiving LOV receptor domain have been identified (Losi and Gärtner, 2008) with PpSB1‐LOV originating from *P. putida* representing a well‐studied example (Drepper *et al*., 2007; Wingen *et al*., 2014). As it is known that marine environments are a rich source for LOV proteins (Pathak *et al*., 2012), we investigated the genomes of the marine *P. pertucinogena* bacteria for occurrence of short LOV proteins using a BLASTP search for homologues of PpSB1‐LOV (NP_746738.1). We identified proteins with >63% identity in every marine strain with exception of *P. litoralis*. The highest identity observed was 75% for a *P. aestusnigri* protein. While it is reported that short LOV proteins occur in 10% of all *Pseudomonas* species generally (Rani *et al*., 2013), this seems to be a remarkable frequency. Hence, the *P. pertucinogena* lineage may represent a promising source for novel FbFPs with unique properties given the fact that these bacteria can thrive in dark, cold and toxic environments.

## Secondary metabolites and storage compounds

*Pseudomonas* species, in general, produce a number of well‐studied secondary metabolites, for example rhamnolipids, phenazines, pyoverdines or syringafactins (Laursen and Nielsen, 2004; Visca *et al*., 2007; Burch *et al*., 2014; Tiso *et al*., 2017). In contrast, the secondary metabolism of the *P. pertucinogena* members still remains undiscovered, despite the eponymous pertucin produced by *P. pertucinogena* that was described to be active against phase I *Bordetella pertussis* (Kawai and Yabuichi, 1975). We therefore mined the genome data available *via* Genbank (Table 1, column 6) of the marine *P. pertucinogena* lineage organisms with respect to secondary metabolite production pathways applying the antiSMASH pipeline with enabled cluster finder algorithm (Weber *et al*., 2015; Blin *et al*., 2017). Not surprising, large gene clusters encoding modular polyketide or non‐ribosomal peptide synthetases (NRPS) were very rarely detected within these comparably small‐sized genomes. Only in the genome sequences of *P. sabulinigri,* an NRPS cluster was predicted that contains four adenylation domains suggesting the synthesis of a hitherto undescribed tetrapeptide derivative. Furthermore*, P. sabulingri* as well as *P. salegens* and *P. pelagia* strain 58 possess putative biosynthesis pathways for aryl‐polyenes, a widespread class of antioxidant natural products (Schöner *et al*., 2016), which, however, seems to be of less importance for biotechnological applications.

The aforementioned capability of *P. pertucinogena* for pertucin production suggests that bacteriocin production might also be found as a feature of these bacteria. Bacteriocins are ribosomally produced peptides which are post‐translationally processed to become antimicrobial peptides and may thus be applied by the pharma or food industries (Hassan *et al*., 2014; Yang *et al*., 2014). Bacteriocin clusters were indeed predicted in the genomes of several species (*P. sabulinigri. P. aestusnigri, P. pachastrellae, P. pelagia* CL‐AP6, *P. oceani*), but they do not appear as a common feature.

In contrast, gene clusters coding for the biosynthesis of the osmoprotectant ectoin are common among the *P. pertucinogena* bacteria and were predicted in all genomes, regardless of whether marine or soil origin. Ectoines are biotechnologically produced, e.g. with *Halomonas spec*., and used as moisturizing ingredients in cosmetics (Yin *et al*., 2015; Bownik and Stępniewska, 2016). Ectoin synthesis is widespread among marine or halophilic bacteria (Yin *et al*., 2015) as it helps to cope with high salt concentrations allowing growth at salt concentrations up to 8%, and in some cases even 15%, as reported for *P. pertucinogena* lineage bacteria (Table 1, column 5).

Notably, several of the investigated genomes seem to contain elements similar to the emulsan biosynthetic pathways. Emulsan is a surface‐active polymeric bioemulsifier best known from *Acinetobacter* species. Bioemulsifiers and biosurfactants are considered as interesting natural products for biotechnological applications as detergents or emulsifiers in consumer products, pharmaceutical or environmental applications (Rosenberg and Ron, 1997; Fracchia *et al*., 2014; Gudiña *et al*., 2016). Biosurfactant or bioemulsifier production would fit the observed surface activity in cultures of *P. pachastrellae* (Antoniou *et al*., 2015) and is furthermore known among bacteria living in oil‐contaminated environments (Satpute *et al*., 2010; Cafaro *et al*., 2013). A MultiGeneBlast using the *Acinetobacter iwoffii* emulsan cluster (Acc. No. AJ243431.1) as input sequence (Medema *et al*., 2013) revealed that none of the marine strains contains a complete cluster; furthermore, all species lack a protein homologous to the respective polymerizing enzyme Wzy. These strains also lack lipopeptide‐related non‐ribosomal peptide synthetase (NRPS) clusters as well as operons with homology to the rhamnolipid synthesis genes *rhlAB* from *P. aeruginosa*; hence, the biosurfactant production capacities of the *P. pertucinogena* bacteria remain undiscovered.

Bacterial carbon storage compounds, namely triacylglycerols or wax esters and polyhydroxyalkanoates (PHA), are also of interest for biotechnology (Alvarez and Steinbüchel, 2002; Steinbüchel and Lütke‐Eversloh, 2003) with the latter compounds discussed as a naturally produced alternative to common petroleum‐derived polyester materials (Narancic and O'Connor, 2017). Gene loci encoding for PHA production are present in all available genomes of the marine *P. pertucinogena* bacteria according to a MultiGeneBlast analysis using the PHA locus of *P. putida* KT2440 from AE015451 as input sequence. This result confirms microscopical observations of PHA granulae within the cells reported for some of the species (Liu *et al*., 2009). Remarkably, no homologous gene clusters were identified in all terrestrial species tested. Noteworthy, all marine species contained homologs to the wax ester or triacylglyceride synthases of *Alcanivorax borkumensis* and *Marinobacter hydrocarbonoclasticus* (Alvarez, 2016). Marine *P. pertucinogena* species may thus be able to adapt their carbon storage metabolite production to the respective environmental conditions.

## Conclusions

Bacterial species belonging to the recently established *P. pertucinogena* lineage are barely explored until today; nevertheless, it appears that they clearly diverge from other *Pseudomonas* species with respect to their metabolism, genome size and, not least, environmental conditions. Notably, by applying bioinformatics tools for genome mining, we discovered that these bacteria hold a high potential for a variety of biotechnological applications. Presumably, these findings will be corroborated by further approaches of whole genome sequencing, *in silico* genome data mining, gene synthesis and expression in established hosts, which will further expand the still limited set of enzymes from already reported but also from other relevant enzyme classes, e.g. keto‐reductases. In addition, the bacteria themselves may increasingly be used for biotechnological applications, in particular, psychro‐ and halophilic as well as hydrocarbonoclastic and heavy metal tolerant bacteria (Margesin and Feller, 2010; Cavicchioli *et al*., 2011; Cafaro *et al*., 2013; Yin *et al*., 2015). The current reports on characterized enzymes are limited to only a few marine species, but it is tempting to speculate that future studies on terrestrial species of the *P. pertucinogena* lineage may uncover such features as well; especially in species as *P. bauzanensis* which can cope with toxic contaminations. *P. pertucinogena* bacteria are easy to cultivate, at least in complex media, and can biosynthesize natural products such as PHAs, ectoin or bioemulsifiers, even with hydrocarbon pollutions or human‐made polymers as alternative carbon sources, thus contributing to the saving of natural resources (Wierckx *et al*., 2015).

## Conflict of interest

None declared.
